# The Effect of Urban Land-Use Change on Runoff Water Quality: A Case Study in Hangzhou City

**DOI:** 10.3390/ijerph182010748

**Published:** 2021-10-13

**Authors:** Li Li, Qidi Yu, Ling Gao, Bin Yu, Zhipeng Lu

**Affiliations:** 1 School of Public Affairs, Zhejiang University, Hangzhou 310058, China; lichs@zju.edu.cn (L.L.); 12022048@zju.edu.cn (Z.L.); 2 School of GeoSciences, University of Edinburgh, Edinburgh EH9 3FE, UK; 3 Wang Yanan Institute for Studies in Economics, Xiamen University, Xiamen 361005, China; 4 Department of Economics, University of Essex, Colchester CO4 3SQ, UK; bin.yu@essex.ac.uk

**Keywords:** land-use change, surface runoff, water pollution, urban facilities

## Abstract

The main functions of this research are to guide the proportion of urban land that is used and the layout of the facilities on it, help understand the changes to surface runoff that are caused by land being used in urban development, and thus solve surface runoff pollution. Hangzhou City, China has been selected for the experiment, and the way in which its land is utilized as well as the grading of urban construction projects in the demonstration area are specifically analyzed. This study systematically distinguishes the definitions of impervious area based on the Sutherland equation and analyzes the impact of different impervious area subtypes on surface runoff water quality. Then, we compare the impact of impervious area subtypes with the impact of other land-use patterns on surface runoff water quality. This study shows the relationship between different land-use types and runoff water bodies: Land-use index can affect runoff water quality; Greening activities, impervious surface, and the water quality index are negatively correlated; the effective impervious area rate is positively correlated with the water quality index. The paper suggests that increasing the proportion of green spaces and permeable roads in build-up land reduces the effective impervious area (EIA) and thus controls land runoff pollution and improves runoff water quality.

## 1. Introduction

Water is mankind’s most important natural resource [1,2,3,4]; it is thus necessary to protect it and avoid any harmful effects endangering our water bodies [5,6,7,8,9]. In line with accelerated global urbanization, the aggravation of water resource destruction needs an urgent solution. Indeed, due to increases in human activities and the expansion of urban land, the quality and quantity of available water resources in populated areas are rapidly declining; thus, the sustainability of water resource management has become more urgent [10,11,12]. Many cities are located on water bodies (lakes, rivers, reservoirs, estuaries, and coastal oceans) and provide important ecosystem services such as drinking water and food provision, water circulation and regional climate regulation and recreational opportunities [13,14]. Various studies have shown that the urban population and population density is rising, and urban buildings are expanding, meaning land reuse and space recombination are eventually leading to changes in the surface structure of cities, breaking the balance of surface radiation, and altering the original urban water cycle and water resource utilization system [15,16,17,18].

Land-use change has a very complex impact on urban water resources. Specifically, rainfall-runoff changes in accordance with rainfall amount and intensity as well as land-use characteristics [19]. The original urban surface has gradually changed from land and sand to hard soil, weakening the heat and water exchange between the ground and the atmosphere [20]. Thus, the amount of rainwater flowing into the subsurface has been reduced, and its water absorption capacity has declined [21]. Moreover, studies have shown that only one-fifth of rainwater is used as runoff in the environmental system as a whole [22]. In highly urbanized areas, urban roads are almost non-permeable to rainwater, and such roads are also increasing, with almost four-fifths impermeable to precipitation [23,24,25]. This spreading of runoff subsequently enters the urban drainage system, and floods generally occur if the amount of rainwater accumulated exceeds the capacity of the urban drainage infrastructure. Meanwhile, rainwater runoff may be polluted by hardened urban road surfaces in built-up areas; The initial rainstorm water that is lost will wash away debris, automobile exhaust emissions, dust, and silt on impermeable roads, such as motorways and open areas. Pollutant sediments are eventually discharged into the water body, and when harmful substances to the human body exceed the range within which tap water can purify itself, the properties of water will be adversely altered [26]. In addition, some researchers point out that other factors relating to urban land can affect water quality, such as erosive rainfall events, heavy metals in surface sediments, natural biological filtration devices, and the landscape pattern of the surrounding zones in the urban area. These water quality studies have identified key variables that affect the physical, chemical, and biological processes of surface water bodies [27,28,29,30,31,32].

We study the impact of land-use on the surface runoff after urban infrastructure construction. The purpose of this research is to understand the urban land-use ratio and facility layout and identify the changes to surface runoff that is caused by land-use in urban development, and hence to solve surface runoff pollution. First, we discuss the land-use in terms of the practice of urban planning and design and the influence of land-use change on surface runoff and summarize the key land-use indexes for controlling surface runoff. Second, we explore the best way to solve the quantitative structural relationship within these indexes to control runoff and runoff water quality. The results provide a point of reference for similar places in the future that intend to restrict the number and location of urban facilities in urban development and, therefore, effectively manage the water ecological cycle and water resource utilization.

## 2. Materials and Methods

### 2.1. Study Area

**Figure 1:**
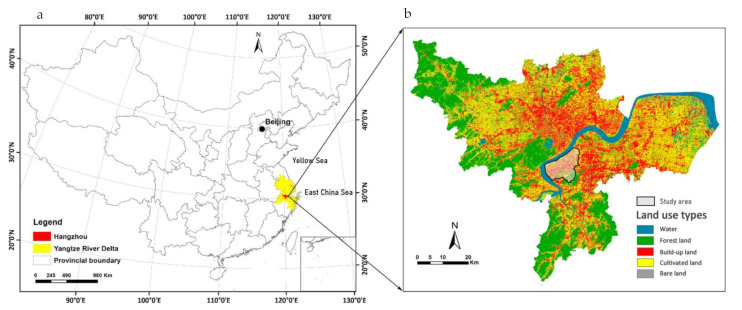
(**a**) Map of China, (**b**) location and land-use types of the study area. Note: The study area is a National High-Tech Industrial Development Zone, and the degree of urbanization has been already very high. In Figure 1b, more than 90% of the cultivated land in the study area is regarded as park land for unplanned built-up land by the government and has been covered by landscape plants such as lawns and trees. It is thus regarded as park land in this study too. The information of Figure 1b comes from City University of Hong Kong.

Hangzhou City is located south of the Yangtze River Delta in China and west of the East China Sea. The latitude and longitude boundary of the city area is 29°11’ to 30°34’ north; 118°20’ to 120°44’ east. The eastern part of the city situated in the North Zhejiang Plain (which forms the Hangjiahu Plain and the Ningshao Plain). The terrain is low and flat, with an elevation of only 3–6 m; There are also dense river networks and lakes. In terms of the city’s land mass, mountains and hills account for 65.6%, plains account for 26.4%, and various water bodies account for 8%. The study area, which is characterized by built-up land, cultivated, and small-scale forest land with densely distributed stream networks, is to the south of the Qiantang River (Figure 1). During the past decade, it has experienced intense urbanization as a large-scale land-use transformation has taken place, meaning croplands and wetlands have been changed into built-up areas.

### 2.2. Runoff Data Acquisition

**Figure 2:**
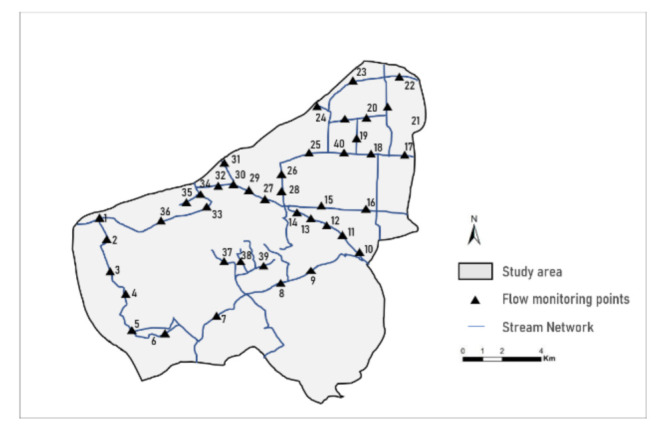
Spatial distribution of surface water quality monitoring points.

The measurement method has been applied to the collection of runoff data and runoff water sample data. Sample data were collected in the study area of Hangzhou City at rainwater outlets because rainwater runoff is representative of the total runoff from different catchment areas, and mirrors land-use. In relation to the runoff into the river, a flowmeter was installed at the outlet of each sampling point to continuously obtain the hourly rainwater runoff. Meanwhile, 2–3 connected tanks were selected as runoff sampling points in each sampling point [33,34]. After the formation of surface runoff, the rainwater collector gathers surface runoff samples every 10 min, 5 times per hour. Then, 40 flow monitoring points and 45 water quality sampling points were set up for this study, 5800 pieces of runoff data and 1400 pieces of water quality sampling data were obtained (Figure 2). All of the data needed to be dimensionless to avoid related deviations being caused in statistical results. 

We assume that there are significant differences in the impact of different types of land-use indicators on runoff water quality. Therefore, according to the river network and the spatial distribution of land-use, this study selected sampling points to evenly cover the spatial distribution of different land-uses, including public building land (i.e., artificial structures such as roads, sidewalks and industrial areas), park land, and unmodified land (e.g., small-scale forest). Samples that were obtained include public construction land samples (R01-R06), park samples (R07-R10), and unmodified samples (U01-U10). Furthermore, to avoid the influence of different basins and divergent rainfall levels, the runoff modulus coefficient was selected to reflect the size of the runoff, which can be transformed into a dimensionless runoff coefficient; this means the runoff and rainfall can be considered in relation to one another [35,36,37]. Due to the correlation, diverse types of rainfall were selected to be examined. Here, different indexes, including Suspended Solids (SS), Biochemical Oxygen Demand (BOD5), Chemical Oxygen Demand (COD), Ammonia Nitrogen (NH3-N), and Total Phosphorus (TP) were selected to reflect the degree of runoff pollution. The Grubbs test can screen and eliminate the small anomalies below the water level, and the average content of each index in terms of rainfall-runoff is finally obtained. The water quality parameters of the runoff water samples within the study area are shown in Table 1 [38,39].

### 2.3. Acquisition of Land-Use Data

The urban land-use situation in 2020 was drawn using computer-aided design (CAD) ground buildings and ArcMap, and the urban base coverage information was obtained through statistical data and manual interpretation powered by ENVI. According to foreign concepts of building permeability and effective impervious area, land-use structure can be divided into effective impervious area (EIA) and ineffective impervious area (IIA). IIA includes green space and artificial permeable surface. The artificial permeable surface includes the roof green design, permeable road, and artificial water bodies. EIA refers to the impervious area that is directly connected to the drainage collection system through rainwater pipelines [40,41]. When compared with impervious surfaces, EIA contributes more to urban area runoff. IIA is a city facility in the study area that cuts off drainage collection systems in impervious areas. Effective impervious area and ineffective impervious area jointly constitute the total impervious area (TLA).

### 2.4. Land-Use Classification

**Figure 3:**
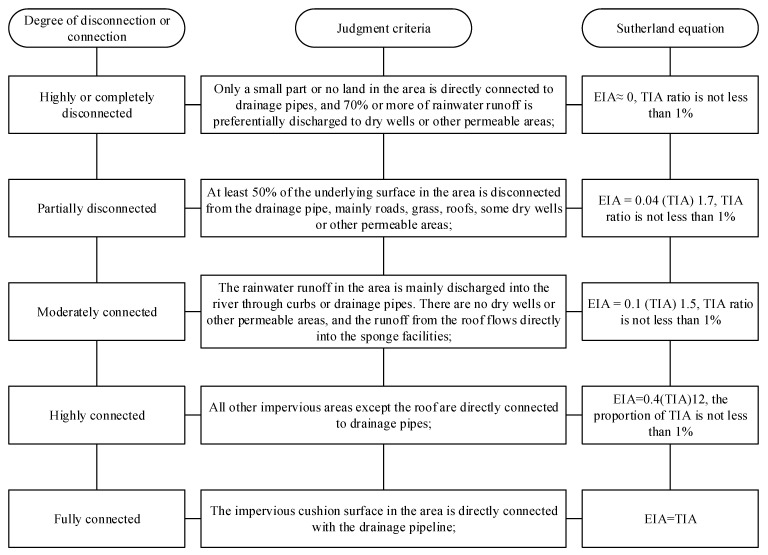
Effective impervious area (EIA) obtained by Sutherland equation.

To determine the influence of land-use on surface runoff and runoff water quality, the scope of the research should first be determined. In accordance with the development trend of watershed systems, the water-quality grade of an area’s runoff and the first-level runoff are selected for watershed division [42,43]. Hangzhou City is a plain river-network city, and the drainage network of different land types are directly connected with the river, so each plot includes a separate watershed. Here, the value of land-use zoning in different regions are obtained with the Sutherland classical equation; the Sutherland classical equation can calculate the site effective impervious area rate, which is connected to the issue of small watersheds (8–28 hectares) according to the Chinese Geological Survey (the specific method is shown in Figure 3). The sample points for transformation are determined based on the construction drawings of water absorption facilities at each point, and then they are combined with what needs to change in line with the classification criteria in Figure 3 [44,45]. The anti-seepage area of residential and multi-person buildings was partially disconnected from the drainage collection system, the anti-seepage area of the park was largely disconnected from the drainage collection system. Lastly, the anti-seepage area in each block was connected to the drainage collection system.

## 3. Results and Discussion

### 3.1. Redundancy Analysis Results

**Figure 4:**
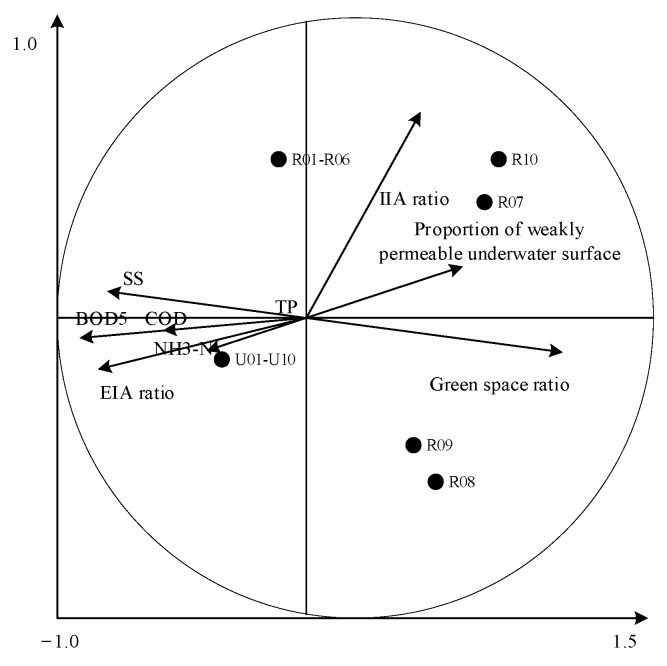
Redundancy analysis of land-use index and water quality indexes.

The sample data redundancy was analyzed with CANOCO5.0 software (WUR, Wageningen, The Nederland). First, the trends found when investigating the result can determine the linear relationship between the dependent variable (water quality index) and the independent variable (land-use index). If the gradient value of the first axis in the four axes is greater than 4, the corresponding analysis model is selected; if the gradient value is less than 3, redundancy analysis is selected; if the gradient value falls between 3 and 4, then both methods then became applicable. After calculation, the slope of the first axis is 0.498, which is far less than 3, indicating that the water quality index is linear with the land-use index [46,47]. Consequently, a redundancy analysis method is selected, and the relationship between land-use and water quality are shown as the arrow in relation to the land-use index and its water quality index (Figure 3), the length and angle of an arrow in Figure 4 indicate that water quality indexes have different responses to land-use indexes [48]. The longer the length of the arrow is, the greater the angle is (a sharp angle means that land-use is positively correlated with water quality indexes, while a blunt angle means there is a negative correlation). The higher the value, the greater the correlation between the two, vice versa.

Figure 4 shows the fact that the types of land-use indexes that are affecting the runoff water quality of each sample are significantly different. The reconstructed public building samples (R01–R06) are mainly affected by the IIA ratio. Park samples (R08 and R09) and park samples (R07 and R10) are significantly affected by the green space ratio and IIA ratio. Unmodified samples (U01–U10) are generally affected by the green space rate and EIA of impervious surfaces. The EIA of construction land is the main index of increased runoff pollution load. Meanwhile, the green space rate, the artificial permeable surface rate, and the IIA are negatively correlated with the water quality index [49]. The correlation between the green space rate and water quality index is the greatest, indicating that the degree of interception of green space on runoff pollution load is higher than the other two indexes. Land use had significant effects on SS, BOD, COD, and NH3-N, while TP had no significant effect on land use. The correlation between the EIA ratio and water quality, and between the IIA ratio and water quality indexes, are compared. The results show that the EIA ratio and IIA ratio have oppositional impacts on water quality indexes, and the correlation between EIA and water quality indexes is higher than that found with regard to the IIA.

### 3.2. PLS (Partial Least Squares) Regression Analysis Results

The PLS regression model is established with SIMCA-P software (Sartorius, Paris, France), and the model is adapted through automatic adaptive analysis. Two local least squares components are extracted with the cross-validation principle within the software. The information utilization rate of the X model is R^2^X (cum) = 0.942, and the interpretability of R is R^2^Y (cum) = 0.828. The cross-validity value Q^2^ (cum) = 0.7819, Q^2^ (cum) is cross validity, indicating that the PLS regression model has a high level of prediction accuracy for new data. Q2 (cum) > 0.5 also suggests that the model has high level of prediction accuracy. When Q^2^ (cum) < 0.05, the model is not significant.

**Figure 5:**
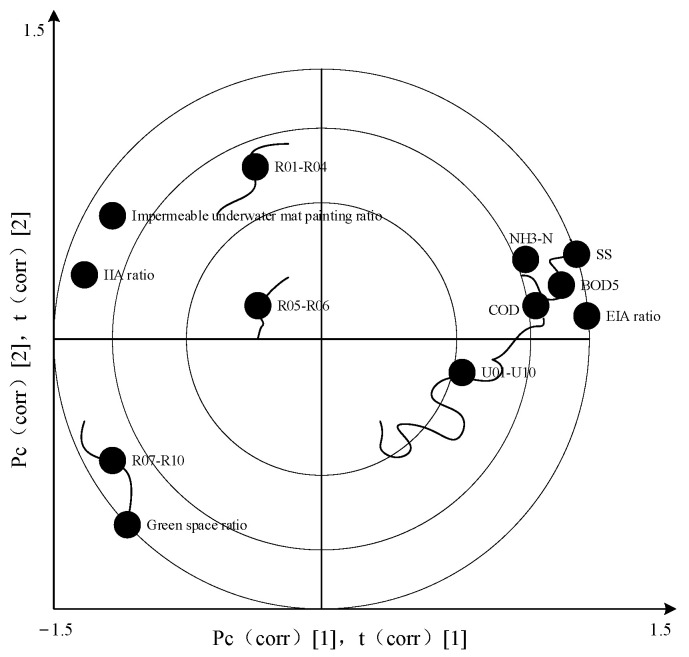
Impact load diagram of land-use types on different drainage zones.

Figure 5 illustrates that the updated plots are mainly affected by the ratio of green space, IIA, and artificial permeable surface. R01–R04 is mainly affected by the proportion of IIA in impervious surfaces, R07–R09 is mainly affected by the proportion of green space. The representative water quality indexes are SS, BOD5, COD, and NH3-N, which are influenced by the effective impervious area.

Some of the empirical equations for the ratio of water quality indexes to land-use indexes are calculated through PLS regression. These equations are greatly affected by soil types. The specific calculation is expressed as in Equation (1).


(1) $$f\left(x\right)=\alpha_{p}+\sum_{p=0}^{n}\left(\mu_{p}\times \beta_{p}\right)$$

In Equation (1), $f\left(x\right)$ stands for water quality index concentration (mg/L), $\alpha_{p}$ represents a constant of *p*, which is the land-use index on water quality indexes, $\mu_{p}$ denotes the level *p* land-use impact index, and $\beta_{p}$ is the *p* land-use index (%).

**Figure 6:**
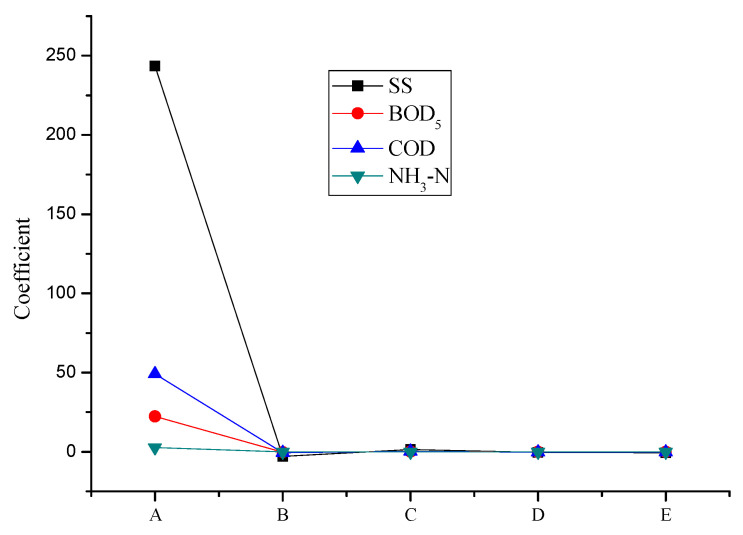
The influence coefficient of land-use on water quality indexes. Note: A—Constant; B—Green space ratio; C—EIA ratio; D—Proportion of artificial permeable surface; E—ineffective impervious area (IIA) ratio.

The degree of influence of different land-use indexes on water quality indexes is shown in Figure 6, and the influence order of their respective impacts is as follows: green space rate > EIA ratio > IIA ratio > water seepage control rate. Specifically, SS is significantly affected by four different land-use indexes, and BOD5 and COD are mainly affected by the green space ratio and EIA ratio; the changes to the amount of NH3-N that are caused by the land-use index are less significant than the alterations that are caused by other water quality indexes, indicating that land-use index is not the most significant index affecting the average concentration of NH3-N [50]. Generally speaking, the higher the EIA ratio, the higher the average concentration of pollutants and the worse the water quality. Additionally, the greater the percentage of green space, the worse the permeability of the IIA water surface. The lower the average content of pollutants that are harmful to the human body, the better the water quality. When rainfall occurs, it is difficult to form surface runoff when the rainwater in the inflow areas, such as green spaces and permeable roads infiltrates into the ground. If the average concentration of pollutants is excessive, it will not rush into the river through the drainage network. Although IIA is an impermeable area, it is not connected to drainage pipes or its surface. The runoff mainly flows to nearby water absorption facilities, rather than directly into the drainage ditch; therefore, the EIA rate can be reduced with an increase in the proportion of green space and in the number of permeable roads in construction land and with the introduction of impervious road runoff into sponge facilities, such as green spaces, which control land runoff pollution and improve river-water quality.

**Figure 7:**
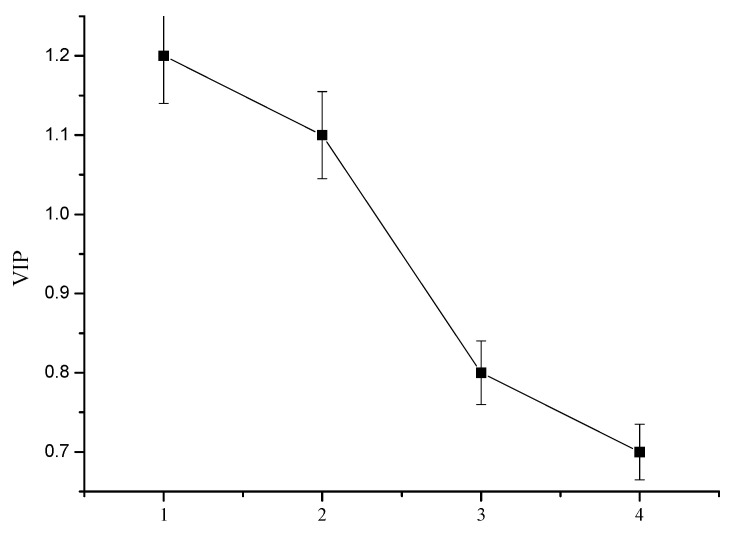
Ranking of variable importance in projection (VIP) values of land-use indexes affecting water quality. Note: 1—Proportion of green space (%); 2—EIA (%); 3—IIA ratio (%); 4—Proportion of impermeable underwater surface (%).

The influence of the land-use importance index on the water quality index can be expressed as a variable importance in projection (VIP) (Figure 7). According to the Figure 7, the VIP value of green space (EIA) is greater than 1, the characteristic ratio is between 0.8–1, and the ratio of green space to the impervious surface is less than 0.8, indicating that green space EIA does affect water quality index, which has important explanatory significance. The green space EIA and the characteristic ratio are the key indexes affecting water quality.

### 3.3. Origin Fitting Prediction Results

**Figure 8:**
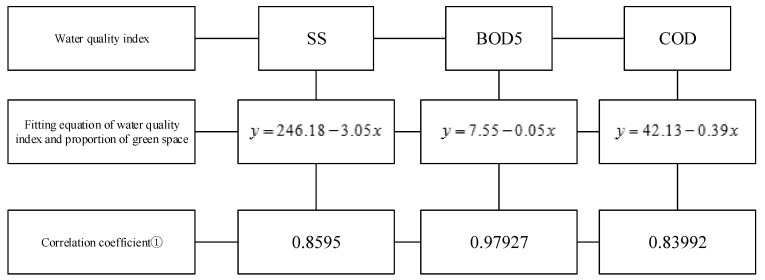
Fitting equation of runoff water quality. Note: ① the closer the correlation coefficient is to 1, the closer the fitting result is to the data to be fitted, and the better the fitting result is. Besides, When EIA ≈ 0, the regression predictions for suspended solids (SS), biochemical oxygen demand (BOD5), chemical oxygen demand (COD), and the proportion of green space.

To control the relationship between land-use types and surface runoff water quality, the fitting relationship between the two is established using the original point fitting equation, and the surface runoff water quality is envisaged in advance (Figure 8).

In line with the analysis of the results of the PLS regression method, the EIA ratio on the *X*-axis and the average concentrations of SS, BOD5, and COD on the *Y*-axis is obtained. According to the disconnection or connectivity between the impervious area of the sample and the drainage network, nine fitting equations are established with Origin2017 software (OriginLab, Northampton, MA, US). The green land is set as the *X*-axis, combined with the other three water quality indexes, and three fitting equations are created. In relation to the obtained sample data, it is predicted that, under the middle gradient of rainfall, the conversion level of different runoff water quality can reach surface water IV, and the scope of secondary standards of sewage discharge should be controlled, along with the land-use indexes.

Land-use patterns can explain the water quality of all of the samples (Figure 4). This confirms that the land-use pattern has a great impact on the water quality of the study area. Although previous studies have focused on seepage prevention and total impervious area (TIA) factors for built-up land, they rarely distinguish between EIA and IIA and discuss the impact of EIA and IIA on water quality respectively [51,52,53,54]. We also discuss the impact of runoff water quality index on different land types, based on the results from the redundancy analysis. For land-use indexes, the EIA rate is significantly and positively correlated with water quality indexes, such as green space, water permeability, and IIA. Meanwhile, the proportion of green space is negatively correlated with each water quality index, and the correlation between green space proportion and each water quality index is the strongest. 

Previous studies failed to analyze the intensity of the impact of impervious area subtypes on the surface runoff water quality and also failed to compare it with other land-use patterns on the surface runoff water quality indicators [55,56]. This research, therefore, conducted a systematic analysis for the subtypes and comparisons of impervious areas. It is worth noting that the definitions of EIA, TIA, and IIA were distinguished according to the Sutherland equation (Figure 3). The PLS analysis shows that the impact of land-use indexes on the surface runoff water quality indexes from large to small is as follows: green space rate > EIA rate > IIA rate of underlying surface > seepage prevention rate. Finally, the origin fitting equation can predict the response threshold of surface water in class IV and the EIA rate of green space, the surface water quality grade, and the secondary sewage discharge standard. Moreover, the comparison between the IIA and EIA has a greater impact on researching the runoff water quality; also, however, a higher the EIA ratio suggests a higher the average concentration of pollutants of runoff water quality. Reducing the EIA ratio in urban land-use is thus beneficial to the runoff water quality. In other words, this study t sheds light on the beneficial impacts of the construction of impervious areas in the process of urbanization.

## 4. Conclusions

We have examined the impact of construction land types on the urban surface runoff water quality and discussed the specific influential mechanisms. The redundancy analysis shows the relationship between land-use types and runoff water bodies. This study found that the land-use index can affect runoff water quality. There is a negative correlation between greening activities, impervious surface, and water quality index; the EIA rate and water quality index are positively correlated. Soil SS, BOD5, COD, and NH3-N are also correlated with the land-use index. The PLS regression analysis shows the impact of areas’ different land-use on the water quality index: the key indexes affecting water quality are the green space rate and EIA rate; the indexes affecting urban water quality are SS and BOD5. 

Therefore, the choice of land-use appears to reveal that the index of built-up land is an important factor that affects the water quality of the study area, including residential and industrial areas as well as roads. The increased built-up areas in the urban area could result in a considerable increase of non-point source pollutants via precipitation runoff [57]. However, a surge in the number of green areas can have a positive effect on water quality. The EIA ratio is one of other important factors for the urban surface runoff water quality. The higher the EIA ratio, the higher the average concentration of pollutants and the worse the water quality. The paper proposes that there is a need to reduce the EIA ratio by increasing the proportion of green space and the number of permeable roads within build-up land; it is also necessary to introduce impervious road flow in sponge facilities, such as green spaces, to control land runoff pollution and improve runoff water quality.

Through various analytical methods, including redundancy analysis, PLS regression analysis and origin fitting prediction, we have qualitatively and quantitatively studied the impact of land-use change on the surface runoff of urban small watersheds. Although some advancements have been made here with regard to the topic of interest, there are still some shortcomings that require further research and improvement. During future research, attempts to increase the amount of experimental data should be made, the scope of research should be expanded, and ways to make the results of the calculation model more accurate should be adopted. The impact of underground runoff is complex. Here, the land-use change has been only studied from the perspective of underlying surface permeability. Nonetheless, we are aware of other land-use change indexes that do not consider the use of permeable pavements, which may lead to the impermeable facilities being classified into the same underlying surface. Therefore, the analysis index of the underlying surface could be further improved, which would lead to more accurate statistical results.

## Figures and Tables

**Table 1:** Descriptive statistics of the runoff water quality parameters.

Indicator	Maximum	Minimum	Mean (Std.)
TP (mg/L)	2.5	0.16	0.56 (0.82)
SS (mg/L)	62.75	11.63	28.52 (11.86)
BOD5 (mg/L)	3.1	1.1	1.6 (0.39)
COD (mg/L)	24.3	5.05	9.17 (2.73)
NH3-N (mg/L)	0.62	0.04	0.19 (0.11)

Note: TP: Total Phosphorus; SS: Suspended Solids; BOD5: Biochemical Oxygen Demand; COD: Chemical Oxygen Demand; NH3-N: Ammonia Nitrogen.

## Data Availability

The dataset used in this research are available upon request from the corresponding author.
